# Murine Cytomegalovirus Immediate-Early 1 Gene Expression Correlates with Increased GVHD after Allogeneic Hematopoietic Cell Transplantation in Recipients Reactivating from Latent Infection

**DOI:** 10.1371/journal.pone.0061841

**Published:** 2013-04-15

**Authors:** Senthilnathan Palaniyandi, Sabarinath Venniyil Radhakrishnan, Fridrik J. Karlsson, Karen Y. Stokes, Nicolai Kittan, Elisabeth Huber, Gerhard C. Hildebrandt

**Affiliations:** 1 Department of Medicine, Division of Hematology and Oncology, Feist-Weiller Cancer Center, Louisiana State University Health Sciences Center Shreveport, Shreveport, Louisiana, United States of America; 2 Department of Medicine, Division of Hematology and Hematologic Malignancies, University of Utah School of Medicine, Salt Lake City, Utah, United States of America; 3 Department of Molecular and Cellular Physiology, Center for Molecular and Tumor Virology, Louisiana State University Health Sciences Center Shreveport, Shreveport, Louisiana, United States of America; 4 Department of Pathology, University of Regensburg Medical School, Regensburg, Germany; St. Jude Children's Research Hospital, United States of America

## Abstract

The success of allogeneic (allo) hematopoietic cell transplantation (HCT) is limited by its treatment related complications, mostly graft versus host disease (GVHD) and fungal and viral infections. CMV reactivation after HCT has been associated with increased morbidity and mortality, and a causal relation between GVHD, immunosuppressive therapy and vice versa has been postulated. Using a low GVHD severity murine HCT model, we assessed the role of MCMV reactivation and GVHD development. BALB/c mice were infected with either murine CMV (MCMV) or mock and monitored for 25 weeks to establish latency, followed by sublethal irradiation conditioning and infusion of bone marrow plus splenocytes from either syngeneic (syn) BALB/c or allo B10.D2 donors. Engraftment of allo donor cells was confirmed by PCR for D*2Mit265* gene product size. Day+100 mortality and overall GVHD severity in allo MCMV pre-infected recipients was higher than in allo mock controls. Pathologic changes of lung and liver GVHD in immediate-early gene 1 (IE1) positive recipients were significantly increased compared to mock controls, and were only slightly increased in IE1 negative. No significant gut injury was seen in any group. Aggravated lung injury in IE1 positive recipients correlated with higher BAL cell counts both for total cells and for CD4+ T cells when compared with mock controls, and also with protein expression of lung IFN-gamma and liver TNF. No evidence for CMV specific morphologic changes was seen on histopathology in any organ of IE1 positive recipients, suggesting that CMV reactivation is related to increased GVHD severity but does not require active CMV disease, strengthening the concept of a reciprocal relationship between CMV and GVHD.

## Introduction

Human cytomegalovirus (HCMV) belongs to the group of herpesviruses, and the majority of the human population will be exposed to CMV with a prevalence of more than 50% [1]. HCMV has the ability to establish a latent infection in the host after recovery from acute infection, allowing for a lifelong persistence of the virus in the host along with the risk for viral reactivation into the replicating state, HCMV viremia and disease at later time points [2]–[3]. Clinically, severe HCMV disease is rarely seen in the healthy individual, but HCMV still poses a significant risk for morbidity and mortality in the immune-compromised host [4].

Allogeneic hematopoietic cell transplantation (HCT) is a potentially curative treatment option for a variety of hematological malignancies, immunodeficiencies and metabolic storage diseases. Improvements in immunosuppressive therapy, anti-infectious prophylaxis, infection management and better care during long term follow-up have significantly improved HCT outcome [5]–[6].

Nevertheless, HCMV remains a significant cause of morbidity and mortality after allogeneic HCT [7]. CMV pneumonitis, colitis and hepatitis are potentially lethal [8], but have significantly decreased in their incidence since strategies to monitor for CMV reactivation following transplant and preemptive therapy have been employed as standard clinical practice [9]. A reciprocal relationship between viral replication and the development of acute graft versus host disease (GVHD) has been recently reported by Cantoni et al., [10], when GVHD and related immunosuppressive therapy increased the risk of HCMV replication, and when risk for acute GVHD development was augmented during HCMV replication. However, the same was not observed by Wang et al., [11], and respective prospective clinical and experimental studies are still pending.

Over the last decade, murine CMV (MCMV) has been well characterized as sharing strong similarities to HCMV [12]–[13]. Following MCMV infection of naïve mice, latency is established in various organs after different time points (spleen: 1–2 months; lungs: 3–4 months; salivary glands: 5–6 months) [14]. The cellular mechanism underlying MCMV viral reactivation is still not completely understood [15]. Previous studies suggested that reactivation is initiated by transcriptional activation of MCMV immediate-early (IE) genes, as they are the first to be detected during reactivation [16].

Using a murine HCT model, in which GVHD develops across minor histocompatibility antigen (mHag) mismatches, we now tested, whether severity of GVHD and HCT outcome are altered in latently MCMV infected recipients. Overall survival was decreased in allogeneic recipients, and MCMV reactivation determined by the expression of IE1 [17] occurred after HCT in the absence of medical immunosuppression and was linked to increased GVHD target organ injury.

## Materials and Methods

### Mice and MCMV infection

Ethics Statement: All experiments were approved by the Institutional Animal Care and Use Committee (IACUC) of Louisiana State University Health Sciences Center, Shreveport, LA; protocol # P-10-053.

Female BALB/c (*H2^d^*) and B10.D2-*Hc^1^ H2-T18^c^*/nSn (*H2^d^*) mice were purchased from the Jackson Laboratories (Bar Harbor, ME). Prior i.p. injection, frozen MCMV (Smith strain) was thawed and diluted in sterile PBS. Recipient mice were infected with either MCMV (3×10^4^ PFU) or mock and then housed in microisolator cages with food and drinking water ad libitum. Following infection, the body weight was monitored weekly until transplantation. After 25 weeks, serum of MCMV infected animals was obtained and tested for the presence of MCMV antibodies by SMART ELISA using SMART-M24 kit (Biotech Trading Partners, USA) according to the manufacturer's protocol.

### Hematopoietic cell transplantation (HCT), clinical GVHD assessment and survival

Latently MCMV infected or mock treated BALB/c recipient mice were sublethally (750cGy TBI single dose) irradiated using cesium source irradiator prior to the tail vein infusion of 7×10^6^ bone marrow cells, and 3×10^6^ splenocytes from either syngeneic (BALB/c) or allogeneic (B10.D2) donor mice. Transplant parameters were chosen to avoid excessive acute GVHD-related mortality in allogeneic recipients so that sufficient animals would be available for analysis at day +100 after HCT. Survival was monitored daily until day +100 after HCT, and clinical GVHD scores were assessed weekly using a commonly used acute GVHD scoring system incorporating following five clinical parameters:, weight loss, posture (hunching), mobility, fur texture and skin integrity [18]. Each parameter was graded between 0 and 2. Once an animal reached a cumulative score of more than 6.5 or a weight loss of more than 30%, it was sacrificed and counted as death due to transplantation related mortality.

### Histopathology

At 100 days after transplantation, animals were sacrificed for analysis. Organs were removed and fixed in formalin for 48 hrs, then transferred into 70% ethanol, paraffin-embedded and sectioned. Hematoxylin–eosin-stained lung, liver and colon sections from individual mice were coded without reference to mouse type and independently examined by a pathologist (E.H.) to establish an index of GVHD injury. Lung tissue was evaluated for the presence of periluminal infiltrates (around airways and vessels) or parenchymal pneumonitis (involving the alveoli or interstitial space), using a modified semi-quantitative scoring system that incorporates both the severity (score 0–3) and extent (percentage of lung space involvement) of disease [18]. Histopathologic changes of the liver were assessed in a semi-quantitative manner by analyzing 9 features that were graded from 0 (normal), 0.5 (focal and rare), 1 (focal and mild), 2 (diffuse and mild), 3 (diffuse and moderate), 4 (diffuse and severe) [19]–[20]. Histopathology scoring of the colon was performed as described previously [21]. Slides were assessed using a Zeiss Axioskop 40 microscope with wide-field eyepiece number 25 (at 400 magnifications, field size of 0.625 mm^2^).

### Broncho-alveolar lavage (BAL) and FACS

At the time of analysis, mice were sacrificed and lungs were lavaged for BAL fluid collection as previously described [18]. In brief, BAL fluid and cells were harvested from the alveolar space through trans-tracheal access. The lavage was done by instilling 1 ml ice-cold PBS into the lung with subsequent aspiration for collection. This was repeated 4 times. Samples were then centrifuged at 1300 RPM for 7 minutes, and the supernatant from the first sample was frozen and stored. Cell pellets from all samples were combined within groups, cells were washed and counted. Cell suspensions were then stained with fluorescent antibodies to cell surface antigens as previously described [22] and analyzed using a BD LSRII flow cytometer (BD Biosciences, San Jose, CA). Following antibodies were used: APC-anti-CD4 (clone RM4-5) (Biolegend, San Diego, CA) and PerCP–anti-CD8 (53-6.7) antibodies (eBioscience, San Diego, CA). Flow cytometry data were analyzed using FlowJo software (Tree Star, Ashland, OR).

### Evaluation of donor/recipient chimerism

Splenic DNA was isolated by using DNeasy Blood & Tissue kit (QIAGEN, Valencia, CA). DNA was checked for quantity and quality in nano drop. *D2Mit265* is a common gene present in both BALB/c and B10.D2 with few amino acid changes. *D2Mit265* gene was analyzed by PCR using primers 5^/^-AATAATAATCAAGGTTGTCATTGAACC-3^/^ and 5^/^-TAGTCAAAATTCTTTTGTGTGTTGC-3^/^ (Integrated DNA Technologies Inc., San Diego, CA) with an expected product of 139 bp for BALB/c and 103 bp for B10.D2. The reaction was formed in following condition: 1 cycle at 95°C for 2 min; 30 cycles of 30 sec at 95°C, 30 sec at 55°C and 30 sec at 72°C; and 1 cycle at 72°C for 5 min. The reaction was performed in total volume of 50 ul with Promega enzyme and reagents, using a PTC-100 (MJ Research, Inc Waltham, MA) thermocycler. Amplified products were separated by electrophoresis in 1% agarose gels, and gels were stained with ethidium bromide.

### MCMV reactivation

DNA was extracted from spleen by use of the DNeasy Blood & Tissue kit (QIAGEN, Valencia, CA). Primers for transcription of IE-1 were used as described [23]. DNA was amplified in the following condition: 1 cycle at 94°C for 3 min; 35 cycles of 30 sec at 94°C, 30 sec at 53°C and 30 sec at 72°C; and 1 cycle at 72°C for 7 min; the reaction was performed in total volume of 50 ul with Promega enzyme and reagents. Amplified products were separated by electrophoresis in 1% agarose gels, and gels were stained with ethidium bromide.

### Measurement of cytokine and chemokine levels by ELISA

Lung, liver and colon samples were homogenized in cold PBS with complete protease inhibitor cocktail (Roche Diagnostics, IN, USA). The levels of TNF, IFN- γ, CXCL1 and CXCL9 were detected using specific standard sandwich ELISA. TNF and IFN- γ were detected using mouse cytokine ELISA kit from BD Biosciences, CXCL1 and CXCL9 were detected using mouse chemokine ELISA kit from R&D Systems Inc. Minneapolis, MN, USA according to manufacturer's protocol. Absorbance was measured at 450 nm using an ELISA plate reader (MULTISKAN FC, Thermo Scientific, Asheville, NC USA).

### Statistical analysis

All values are expressed as the mean ± SEM. Survival curves were plotted and compared by log-rank analysis. Statistical comparisons between groups were completed using an unpaired *t* test. *P*<0.05 was considered statistically significant.

## Results

### Infection with MCMV Smith strain successfully mounts anti-MCMV IgG seroconversion

**Figure 1:**
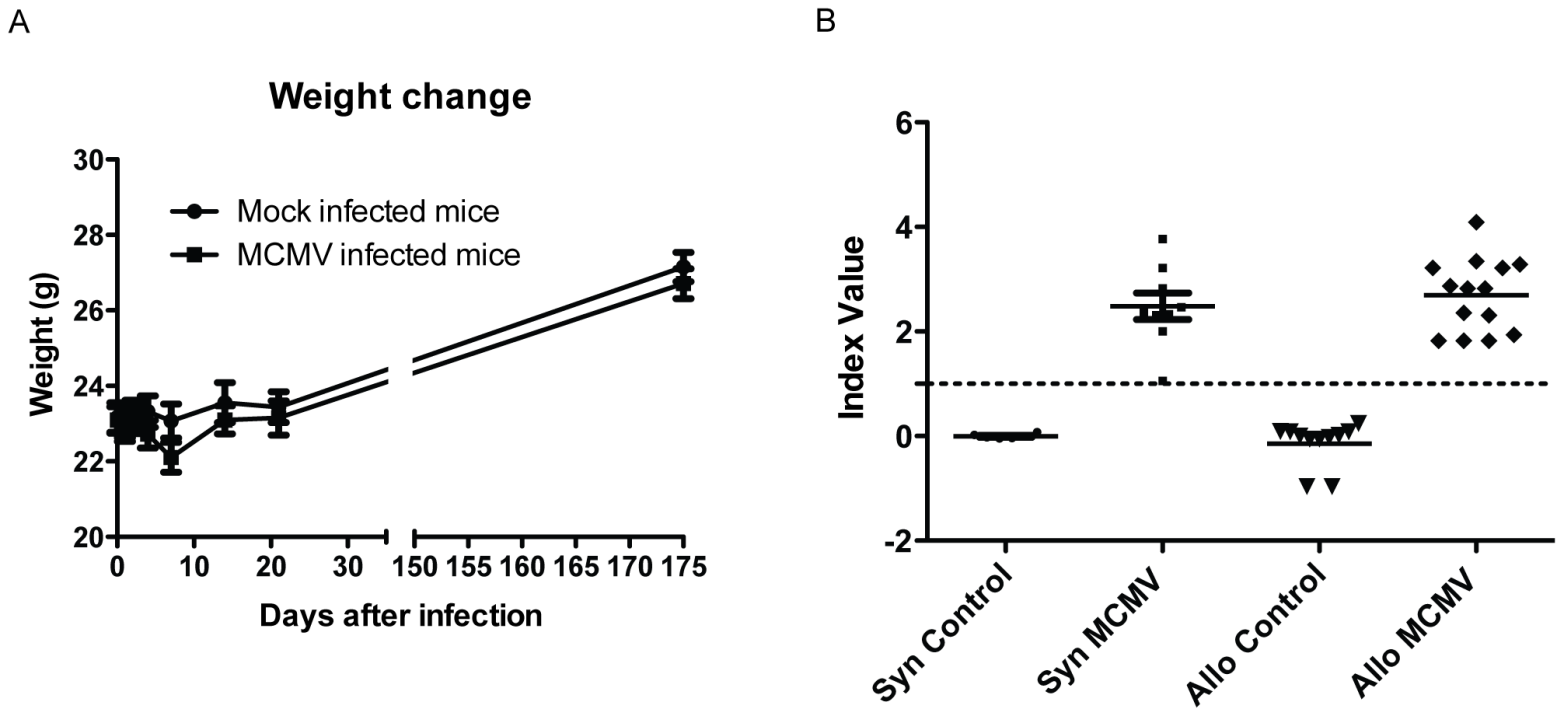
Weight change after MCMV infection and MCMV serology testing. MCMV infection was done by *intraperitoneal* injection of 3×10^4^ PFU purified Smith strain in naive BALB/c mice and another set of mice were mock infected as control. (A) Weight change was monitored following infection for 25 weeks; n per group = 28 (MCMV) and 24 (mock); Data are presented as mean. (B) 25 weeks following infection, animals were analyzed for anti-MCMV IgG seropositivity as indicator for MCMV infection. Data shown present the index value with ≥1 determined as positive, data points for individual mice are shown.

Recipient mice were either infected with MCMV Smith strain or mock treated as described in Materials and Methods. MCMV infection was well tolerated and no MCMV-related death occurred during the observation period of 25 weeks. MCMV treated animals showed no difference in weights and clinical scores when compared to mock infected (Fig. 1A). Prior to subsequent transplant, animals were analyzed for MCMV seroconversion by ELISA in order to ensure successful MCMV infection. As shown in figure 1B, anti MCMV IgG antibodies were detected in all mice treated with the virus, and as expected, none of the mock treated animals was tested IgG positive. None of the animals was clinically sick at this time point and accordingly considered to be latently infected.

### MCMV latency increases clinical GVHD severity and mortality after allogeneic HCT

**Figure 2:**
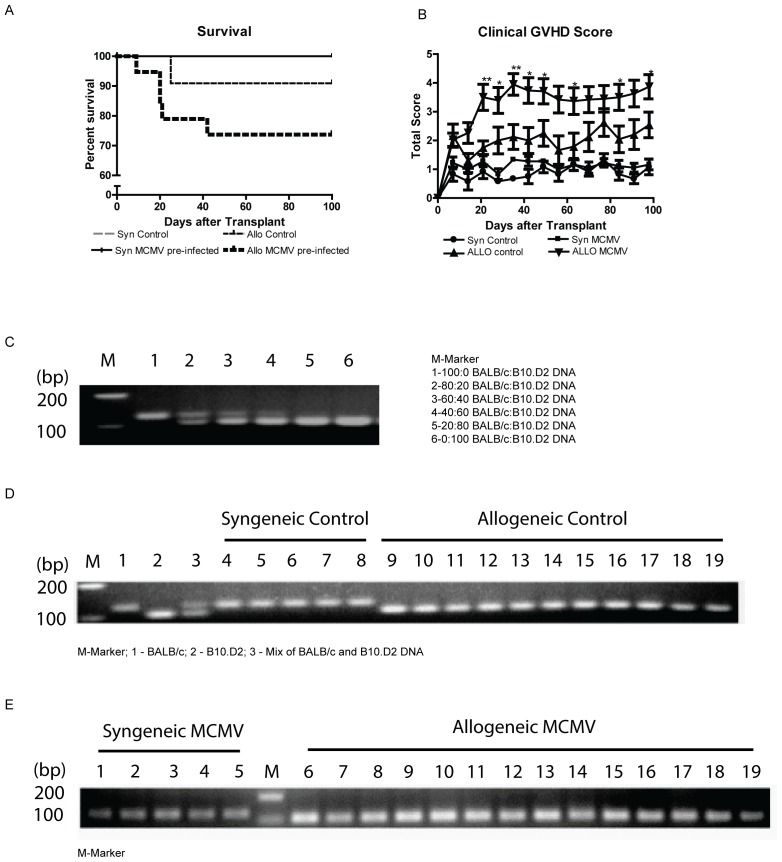
Survival, clinical GVHD and engraftment following HCT. (A+B) Animals were transplanted as described in Materials and Methods, and survival and clinical GVHD scores were monitored for 100 days (*n* = 6 for syngeneic control group; *n* = 9 for the MCMV treated syngeneic group, *n* = 18 for allogeneic control group and *n* = 19 for the MCMV treated allogeneic group). Data are combined from two identical experiments. (*p<0.005,**p<0.001). (C–E) Detection of gene *D2Mit265* PCR products for BALB/c (139 bp) and B10.D2 (103 bp) was used to determine donor cell chimerism in the spleen.

Following a waiting period of 25 week after MCMV or mock infection, animals underwent HCT from either syngeneic (BALB/c) or allogeneic (B10.D2) donors. Hypothesizing, that MCMV potentially exacerbates GVHD, a conditioning regimen of 750cGy TBI and a relatively low dose of 3×10^6^ splenocytes were chosen as transplant parameters. Hereby it was aimed to achieve donor engraftment and at least mild GVHD pathology in allogeneic controls, and at the same time to allow for a sufficient number of allogeneic MCMV treated recipients reaching the planned time endpoint for analysis at day +100. Mice were divided into four experimental groups: group 1 - mock infected syngeneic; group 2 - MCMV latent syngeneic; group 3 - mock infected allogeneic; group 4 - MCMV latent allogeneic. Following HCT, survival and clinical GVHD were monitored daily or weekly, respectively. As expected, all mock treated syngeneic recipients survived and were clinically healthy. MCMV latent syngeneic recipients showed slightly elevated clinical scores initially, but after 8 weeks did not differ from syngeneic Mock controls. Allogeneic controls developed moderate clinical symptoms of GVHD and were evidently sick when compared to syngeneic groups, yet as intended, mortality was still low (8.3%). In contrast, allogeneic MCMV infected animals showed increased mortality (26.3%) along with significantly increased clinical GVHD scores (figure 2A–B).

### Chimerism analysis after allogeneic HCT using *D2Mit265* gene polymorphism

To exclude differences in engraftment of allogeneic recipients accounting for the observed differences between groups, we next tested for splenic donor chimerism in survivors at day +100, by analyzing for *D2Mit265* as described in Materials and Methods. The amplified *D2Mit265* gene product in BALB/c mice is 139 bp of size, where as it is 103 bp in B10.D2 animals. As depicted in figure 2C, mixing studies of BALB/c and B10.D2 DNA show absence of BALB/c 139 bp product size at a ratio of 20 (BALB/c): 80 (B10.D2), and absence of B10.D2 product size at a ratio of 100∶0, respectively. As demonstrated in figure 2D–E, syngeneic recipients showed as expected a product at 139 bp only. BALB/c recipients receiving B10.D2 donor cells demonstrated at least 80% donor chimerism, consistent with successful donor cell engraftment.

### Immediate early 1 (IE1) gene expression as indicator of MCMV reactivation

**Figure 3:**
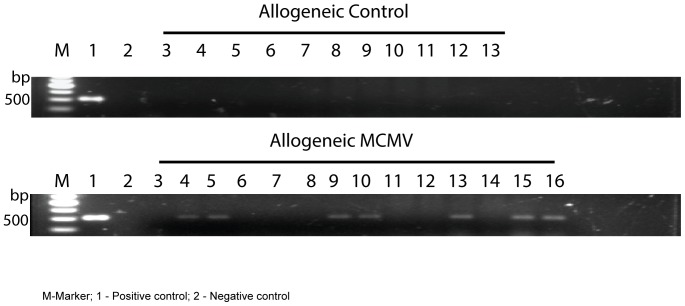
Reactivation of MCMV in latently infected mice. Viral gene IE1 expression was assessed by PCR splenic DNA at day+100 after transplant.

CMV reactivation does not occur in all patients after allogeneic HCT and has been associated with both the presence of acute GVHD and related immunosuppressive therapy. CMV positive patients receiving HCT from a CMV negative donor are at highest risk due to the lack of CMV directed donor memory T cells, which can account for some protective immunity after HCT. Recipients in our model fall into this high risk group for MCMV reactivation, despite no further immunosuppression being given for evolving GVHD. Therefore, CMV reactivation may have occurred in all animals or at different time points during the follow-up period. To assess the contribution of MCMV reactivation to GVHD pathology, we aimed to identify those allogeneic animals active virus at time of analysis by using MCMV IE1 expression in the spleen as a discriminating marker as described in Materials and Methods [24]. IE1 transcripts were not detectable in the mock-infected allogeneic group, whereas they were seen in half of the MCMV allogeneic group (figure 3). On the basis of these results, we decided to split the allogeneic recipients into three groups for future analysis: allogeneic MOCK, allogeneic MCMV IE1 negative and allogeneic MCMV IE1 positive.

### Pulmonary and hepatic injury is aggravated in allogeneic recipients expressing MCMV IE1

**Figure 4:**
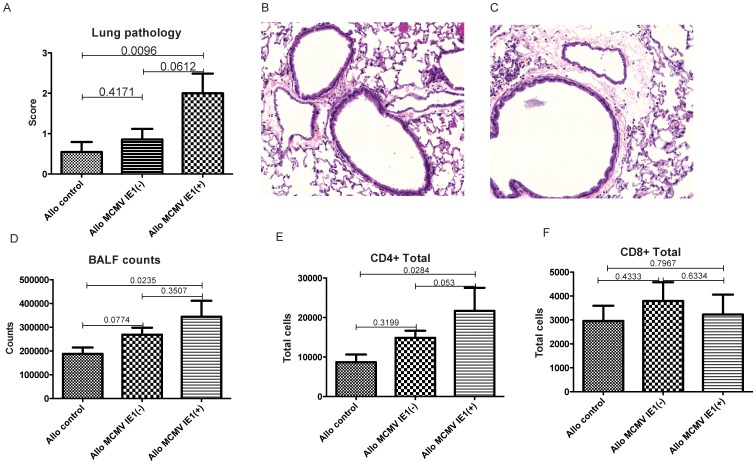
Lung injury after allogeneic HCT. Animals were transplanted as described in Materials and Methods. (A) Lung pathology was semiquantitatively assessed on day +100. Data are expressed as mean ± SEM. n = 11 (mock), 7 MCMV IE1(−) and MCMV IE1(+), respectively. (B)+(C) Example of histopathological changes (H&E stain, magnification: 200×) for (B) mock (normal lung tissue without or with minimal periluminal inflammation around airways and blood vessels, no parenchymal pneumonitis and (C) IE1(+): periluminal inflammation around airways and blood vessels but no major parenchymal pneumonitis. (D–F) Total BALF cellularity and CD4+ and CD8+ BAL T cells at day +100 after HCT.

**Figure 5:**
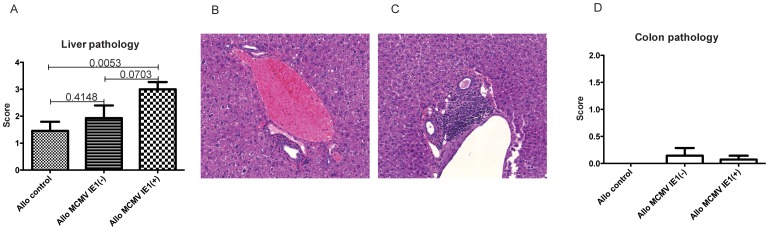
Hepatic GVHD after allogeneic HCT. Animals were transplanted as described in Materials and Methods. (A) Hepatic GVHD pathology was semiquantitatively assessed on day +100. Data are expressed as mean ± SEM. n = 11 (mock), 7 (MCMV IE1 (−)) and (MCMV IE1 (+)), respectively. (B–C) Example of histopathological changes (H&E stain, magnification: 100×) of the liver showing portal tract with moderate lymphocytic inflammation in IE1(+)>mock. (D) Colonic GVHD pathology was semiquantitatively assessed on day +100. Data are expressed as mean ± SEM. n = 11 (mock), 7 (MCMV IE1 (−)) and (MCMV IE1 (+)), respectively.

**Figure 6:**
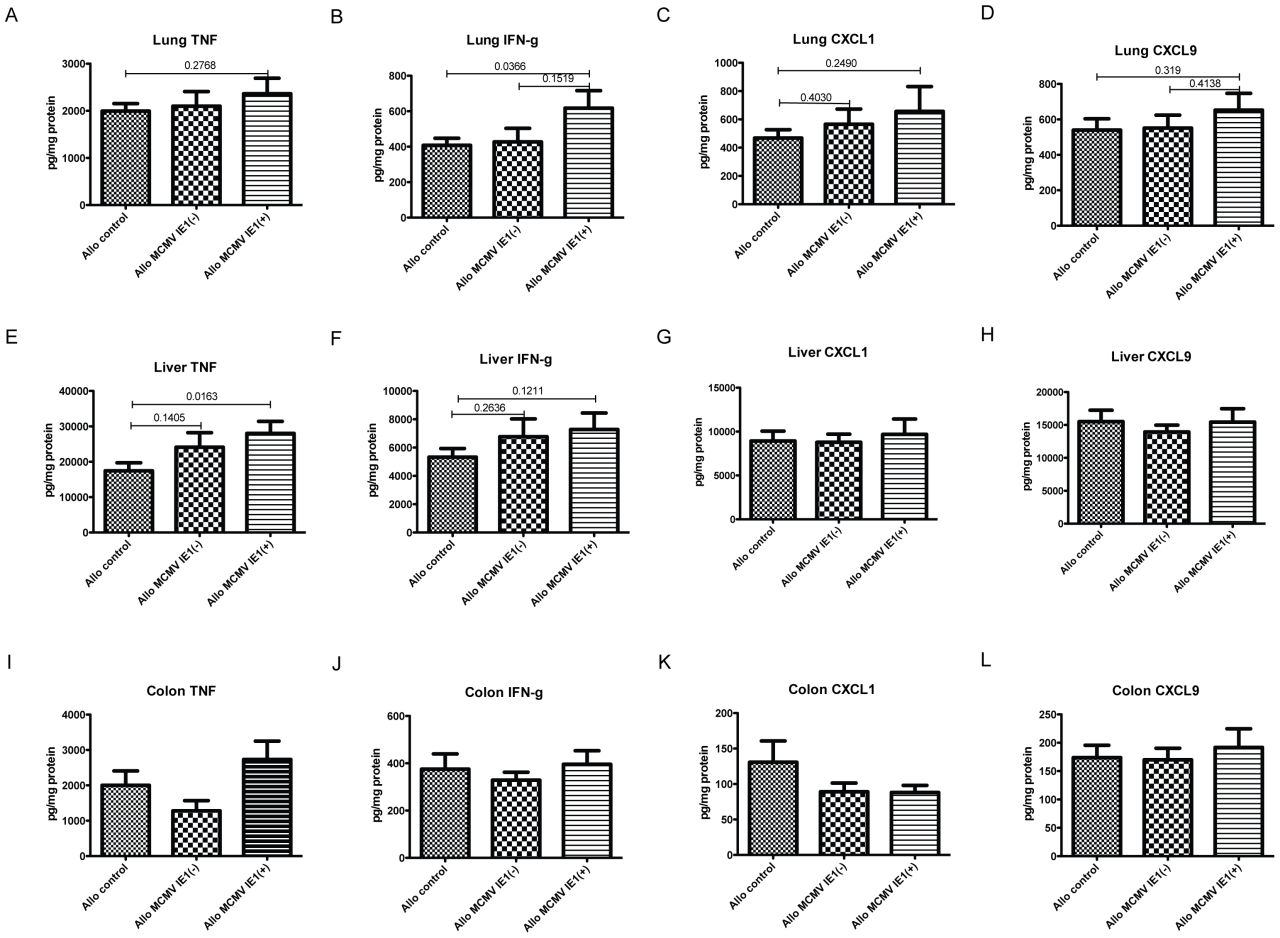
Inflammatory cyto- and chemokine expression in lung, liver and colon: Animals were transplanted as described in Materials and Methods. TNF (A, E, I) IFN-gamma (B, F, J), CXCL1 (C, G, K) and CXCL9 (D, H, L) in organ homogenates were measured by ELISA. Results are shown as mean ± SEM; n = 11 (mock), 7 MCMV IE1(−) and 7 MCMV IE1(+).

Histopathologic changes in the lung were observed in all three allogeneic groups at day +100 after HCT, predominantly presenting as perivascular lymphocytic inflammation and lymphocytic peri-bronchiolitis, but not consistent with the four major patterns of CMV infection of the lung: diffuse interstitial pneumonititis, hemorraghic pneumonia, CMV inclusions associated with minimal inflammation or military pattern. When comparing allogeneic mock treated animals with MCMV latent IE1 negative recipients, pathology scores did not statistically differ. In contrast, IE1 positivity was associated with a significant increase in pulmonary injury when compared to mock controls and with a statistically non-significant increase when compared to IE1 negative recipients (figure 4A–C). Lung pathology in MCMV latent IE1+ allogeneic recipients correlated with elevation of total BAL cells counts and of CD4+ but not of CD8+ T cell counts (figure 4D–F). Exacerbation of GVHD related changes was also seen in the liver of MCMV IE1+ allogeneic recipients (figure 5A–C), whereas no GVHD related injury was found in the colon (figure 5D). Importantly, findings suggestive or characteristic for MCMV disease were not found in either organ. No differences in histopathology were seen between syngeneic groups (data not shown).Lung pathology was associated with increased pulmonary IFNg protein levels (p = 0.0366) as well as trending increases in TNF, CXCL1 and CXCL9 (figure 6A–D). In the liver, no difference was seen for CXCL1 and CXCL9, while IFNg trended to be higher and TNF was significantly elevated in the MCMV IE1+ allogeneic recipients (p = 0.0163) (figure 6 E–H). Consistent with pathology findings, no differences were seen for cyto- or chemokine expression in the colon (figure 6I–L).

## Discussion

Reactivation of CMV from latency results in significant morbidity and mortality in patients after allogeneic HCT. The molecular mechanism by which this occurs is not clear. Previous work has identified the lungs and liver as major organ sites of CMV latency and recurrence in mice [25]–[26]. Induction of IE1 gene expression may be a crucial first step in the reactivation process [27]. A link between CMV reactivation and the increased risk of GVHD has been previously suggested [28]. We now for the first time correlate IE1 gene expression, as indicator for reactivation from latency, with the severity of pulmonary and hepatic GVHD after murine HCT.

CMV latency was established in BALB/c mice by intraperitoneal injection with 3×10^4^ PFU of Smith strain MCMV six months prior to subsequent HCT. This time span was chosen in consistence with prior studies [29]–[30]. Due to the natural course of CMV infection both in mice and humans, CMV seropositivity is commonly used as a surrogate marker for latent infection, associated with an increased likelihood that seropositive allogeneic HCT recipients reactivate CMV latently present in leukocytes and in target organs. However, the association of either donor or recipient CMV seropositivity with GVHD development is not clearly defined [31]–[37]. While GVHD and GVHD therapy-related immunosuppression are broadly accepted as risk factors for CMV reactivation, there is conflicting data from retrospective clinical studies on the inverse causal link between CMV reactivation and induction or aggravation of GVHD [11], [31], [38]. CMV infection or reactivation can precede the development of chronic GVHD and contribute to delayed immune recovery in the post-transplant period [39]. The concept of infections propelling GVHD development is not restricted to CMV, as e.g. Poutsiaka et al. demonstrated that blood stream infections can trigger acute-GHVD complications after HCT [37].

We found, using a murine HCT model that led to donor cell engraftment, that uninfected recipients or those exhibited non-actively replicating virus developed low severity clinical GVHD, whereas recipients with actively replicating MCMV demonstrated more severe GVHD changes in liver and lung. No evidence of CMV disease in these organs was found.

CMV reactivation and associated CMV disease play only a limited role in autologous HCT of seropositive patients [40]. This may be explained by a number of reasons including the more rapid immune recovery following transplant, the absence of GVHD, the lacking need for prophylactic or therapeutic immunosuppressive medication and the reinfusion of the recipient's CMV specific memory B and T cells with the transplant inoculum in seropositive patients [41]. Except for the latter, all is true for syngeneic HCT recipients in this study, and all latently CMV infected syngeneic recipients survived without clinical symptoms and differences in pathology between groups.

The major immediate early proteins exhibit multiple functions, including pro-inflammatory NF-kappa B activation [17], [42]–[43]. IE1 protein of human and murine CMV exhibit same molecular structure [44]. Earlier studies have reported that MCMV IE1 protein transcripts are not detectable in tissues of latently infected mice [29], while IE1 gene expression has been demonstrated to occur during MCMV infection and has been used as a marker for reactivation from latency in the non-transplant setting [45]. Expression of IE1 gene during MCMV reactivation functions as a strong transcription enhancer in the MCMV genome [46]. In this study, we looked at IE1 gene expression in the context of MCMV reactivation after allogeneic HCT. We found that, despite absent histopathologic findings characteristic for CMV disease in target organs, IE1 gene expression was detectable in the spleen of half of the latently infected allogeneic HCT recipients at time of analysis and that those animals demonstrated more severe GVHD injury than IE-1 negative recipients. Interestingly, organ changes of CMV latently infected but IE-1 negative recipients did not differ from allogeneic non-CMV controls, suggesting that clinically relevant CMV reactivation can be identified by the expression of IE-1, potentially promoting GVHD severity.

Prior studies have demonstrated that lungs and liver are the true sites of MCMV latency and recurrence [47], [14], [26]. Consistently, differences in pulmonary and hepatic, but not colonic pathology were seen in our model. Further, CMV has been associated with the development of obliterative bronchiolitis as a form of lung allograft rejection after solid organ transplantation and has been linked to chronic GVHD after HCT, respectively [48]–[49]. Pulmonary findings in our model are not characteristic for obliterative bronchiolitis, though they are consistent with changes seen in chronic lung injury of HCT recipients [50], and it is suggestive, that CMV reactivation indeed is a risk factor for developing this deleterious long-term complication after allogeneic HCT [51].

In summary, our data demonstrate a causal relationship between active replication of CMV in latently infected recipients after allogeneic HCT and the development and aggravation of GVHD. Consequent monitoring for CMV reactivation by quantitative PCR and preemptive treatment in the context of rising viral load are recommended for HCT recipients in the early HCT period, or when developing GVHD or requiring GVHD treatment-related immunosuppressive therapy. The need for these preventive measures shall hereby be reiterated to prevent entry into or acceleration of an evitable circulus virtuosus coagrescendi et moriendi.
